# Body Fat of Basketball Players: A Systematic Review and Meta-Analysis

**DOI:** 10.1186/s40798-022-00418-x

**Published:** 2022-02-22

**Authors:** Pierpaolo Sansone, Bojan Makivic, Robert Csapo, Patria Hume, Alejandro Martínez-Rodríguez, Pascal Bauer

**Affiliations:** 1 Faculty of Sport Sciences, UCAM - Catholic University of Murcia, Murcia, Spain; 2 University of Applied Sciences Wiener Neustadt, Wiener Neustadt, Austria; 3 Centre for Sports Science and University Sports, University of Vienna, Vienna, Austria; 4 Sports Performance Research Institute New Zealand (SPRINZ), Auckland University of Technology, Auckland, New Zealand; 5 Department of Analytical Chemistry, Nutrition and Food Sciences, Faculty of Sciences, University of Alicante, Alicante, Spain

**Keywords:** Fat mass, Team sports, Physique assessment, Skinfolds, Reference values, Playing level, Anthropometry

## Abstract

**Background:**

This study aimed to provide reference values for body fat (BF) of basketball players considering sex, measurement method, and competitive level.

**Methods:**

A systematic literature research was conducted using five electronic databases (PubMed, Web of Science, SPORTDiscus, CINAHL, Scopus). BF values were extracted, with analyses conducted using random-effects models and data reported as percentages with 95% confidence intervals (CI).

**Results:**

After screening, 80 articles representing 4335 basketball players were selected. Pooled mean BF was 13.1% (95% CI 12.4–13.8%) for male players and 20.7% (95% CI 19.9–21.5%) for female players. Pooled mean BF was 21.4% (95% CI 18.4–24.3%) measured by dual-energy X-ray absorptiometry (DXA), 15.2% (95% CI 12.8–17.6%) via bioelectrical impedance analysis (BIA), 12.4% (95% CI 10.6–14.2%) via skinfolds and 20.0% (95% CI 13.4–26.6%) via air displacement plethysmography. Pooled mean BF across competitive levels were 13.5% (95% CI 11.6–15.3%) for international, 15.7% (95% CI 14.2–17.2%) for national and 15.1% (95% CI 13.5–16.7%) for regional-level players. As the meta-regression revealed significant effects of sex, measurement method and competitive level on BF, the meta-analysis was adjusted for these moderators. The final model revealed significant differences in BF between male and female players (*p* < 0.001). BF measured by DXA was significantly higher than that measured by BIA or skinfolds (*p* < 0.001). International-level players had significantly lower BF than national and regional-level players (*p* < 0.05).

**Conclusions:**

Despite the limitations of published data, this meta-analysis provides reference values for BF of basketball players. Sex, measurement method and competitive level influence BF values, and therefore must be taken into account when interpreting results.

## Key Points

- This systematic review and meta-analysis found that body fat of basketball players differs according to players’ sex, competitive level as well as by the measurement method implemented
- Female basketball players have higher body fat than male counterparts. International-level players have lower body fat than national and regional-level players. Across measurement methods, body fat values obtained by DXA are higher than those obtained via BIA and skinfolds.
- Future studies reporting the body fat of basketball players should specify the reliability of measurement, clearly report the hydration and feeding status prior to measurement, specify the competitive level of the sample by reporting the country and/or region and name of the league in which players competed at the time of the study, and report body fat of players in distinct categories (i.e. sex, competitive level, playing position) for better interpretation of data.

## Background

Basketball is one of the most practiced team sports worldwide [1] and has been an Olympic discipline since 1936. The game is characterised by a highly intermittent profile as well as intense neuromuscular actions such as accelerations, decelerations, changes of direction, jumps, lateral sliding and static efforts [2–4]. In basketball, the anthropometric profile of players is a strong performance-limiting factor. Between the mid to late twentieth century, major increases in the average height of players [5, 6] were reported in the U.S. National Basketball Association (NBA), demonstrating that in selection processes more importance was given to the screening of the players’ physical profile.

In many sports, including basketball, body composition is an important feature that is regularly assessed by practictioners [6]. The high locomotion demands of basketball [3] impose considerable physical loads on the players’ bodies [7]; therefore, a more favourable body composition profile (e.g. less fat mass) might be beneficial for the athlete. In fact, the relative proportion of body fat (BF) has been shown to be negatively associated with performance of explosive actions such as changes of direction [8] and vertical jumps [9]. Noticeably, these actions are frequent in basketball (e.g. jumps: ~ 1 ± 0.1 per minute; changes of activity every 1–3 s) [2, 3]. Higher BF has also been shown to increase risk of overuse injuries (e.g. patellar tendinopathy) in basketball and volleyball players [10, 11]. Considering also the high training [12, 13] and competition [12] loads imposed during the basketball season, it appears therefore relevant for basketball practitioners to control players’ BF, in order to optimize their performance and guarantee their health.

With regard to body composition assessments in basketball players, the player’s sex must be taken into consideration. Females possess greater BF content compared to their male peers [2, 14], mainly for evolutionary benefits (e.g. pregnancy) and hormonal differences (higher estrogen) [15]. While this notion is widely known, no study has systematically assessed previous data of BF of male and female basketball players, and thus no precise reference values are available to practitioners yet. This is of foremost importance considering that, to be selected at high levels, basketball players are commonly screened for anthropometric characteristics (including BF) [14, 16] and physical capacities which can be influenced by BF (e.g. jumps, changes of direction) [8, 9].

BF is usually quantified by laboratory (e.g. dual-energy X-ray absorptiometry [DXA], air displacement plethysmography [ADP]) and field methods (e.g. skinfold measurement, bioelectrical impedance analysis [BIA]) all of which have their own advantages and disadvantages [17]. However, it is important to note that each method makes its own assumption when estimating BF, which may yield discrepant results in the same group of athletes.

Furthermore, it is reasonable to expect that BF levels would discriminate players of different competitive levels, since the physiological demands are known to be greater in higher compared to lower leagues [2, 3]. Differences in anthropometric and physiological characteristics, such as body height, aerobic capacity and muscle power have been previously reported, with all parameters favouring players in higher leagues [18–20]. However, in terms of differences in BF the available body of evidence is less clear. For instance, two previous studies [18, 20] reported lower BF content in higher-level players compared to lower levels, two studies found no differences [19, 21], and one study [22] reported higher BF values in national compared to regional-level players.

Reference values for BF in basketball players are needed by researchers, coaches, and practitioners alike when evaluating players. This information should distinguish between female and male players, help interpretation of values obtained by different measurement techniques, and aid in selection processes [16] and training design [23]. Therefore, the aim of this study was to provide reference values for BF of basketball players considering sex, measurement method, and competitive level.

## Methods

### Study Design and Searches

**Fig. 1:**
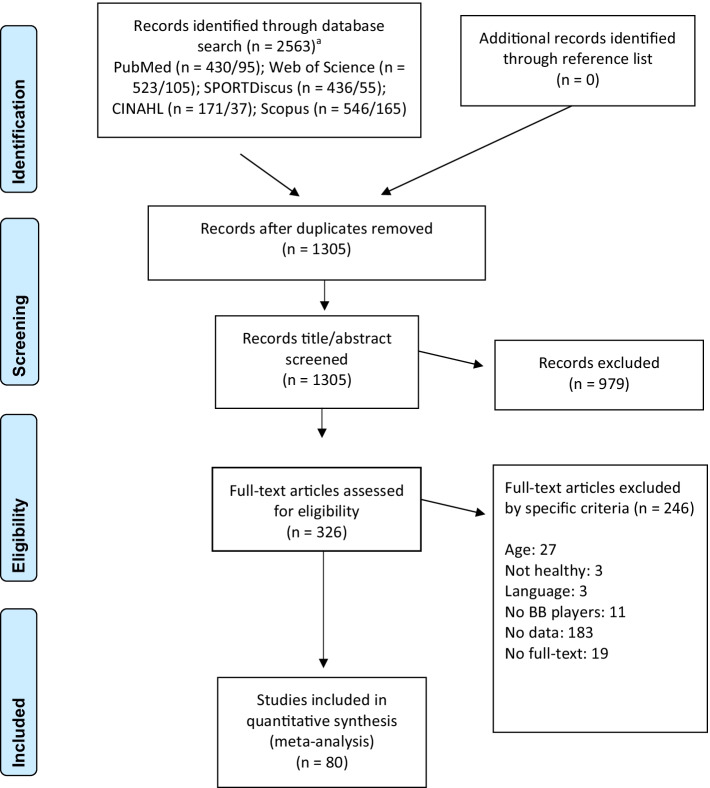
Flowchart of study screening and selection. *BB* basketball. ^a^Data for each database represent results of Jun 2020/Nov 2021 searches

A systematic review and meta-analysis was conducted in accordance with the Preferred Reporting Items for Systematic Reviews and Meta-Analyses statement guidelines [24]. A literature search was performed using electronic databases PubMed, Web of Science, SPORTDiscus, CINAHL and Scopus (Fig. 1). The search was limited to peer-reviewed studies from all languages published between January 2010 to June 2020 and was updated November 2021. The following body composition related search terms were combined with the term “basketball” to source pertinent peer-reviewed articles: “body composition” OR “body fat*” OR “fat mass” OR “lean body mass” OR “fat free mass” OR “muscle mass” OR skinfold* OR anthropometr* OR “multi-component model” OR “bioelectrical impedance” OR bioimpedance OR “magnetic resonance imaging” OR “computed tomography” OR “dual-energy X-ray absorptiometry” OR “dual X-ray absorptiometry” OR densitometry OR “underwater weighing” OR “air displacement plethysmography” OR hydrometry OR ultrasound OR “3D photonic scanning”. The literature search and study selection were independently conducted by three researchers (PS, PB and BM) and disagreements were resolved by discussion until consensus was achieved.

### Study Inclusion and Exclusion Criteria

After database screening and removal of duplicates, the remaining studies were carefully examined by screening the (1) titles, (2) abstracts and (3) full texts. The following inclusion criteria were applied: (1) participants were healthy basketball players older than 18 years; (2) players were competing at regional, national or international competitions; (3) the full-text of the article was published in a peer-reviewed journal in English, Spanish, Portuguese or German language; and (4) outcome measures included and described at least one method of estimating relative BF.

Studies were considered ineligible for this review if (1) the mean age of the sample was lower than 18 years; (2) some or all basketball players were injured (e.g. rehabilitation study); (3) the full-text of the article was not written in English, Spanish, Portuguese or German language; (4) the term basketball player was used referring to athletes from other sports or recreational basketball players, who did not engage competitively, trained less than at least twice per week and/or had less than a minimum of one year of basketball experience; (5) the BF value was not stated, or not independently reported by sex or measurement method, or the study contained duplicate data (e.g. same sample of another study already included in the search results); (6) the article full-text was not available. Case studies, reviews, conference communications, opinion articles, presentations, theses, book chapters or posters were not included. To complement the literature research, the reference lists of the included studies were also screened. The literature review and selection processes are summarized in Fig. 1.

### Data Extraction Strategy

Studies were independently read by three researchers (PS, PB, and BM) for the extraction of the following variables: (1) descriptive information including authors, year of publication and type of study; (2) participant information including sample size, sex, age, body height, body mass and general sample description. Players were assigned to one of three competitive levels: regional, national and international. Players from third national leagues or lower, university athletes or regional teams without further description were considered regional-level, whereas the national level represents players from first or second national leagues, including the National Collegiate Athletic Association (NCAA) divisions 1 and 2. If the study clearly mentioned that players competed at the international level (i.e., members of a national team, club teams competing in international championships) or were playing in the NBA, they were categorised as international level. (3) Measurement information including the technique and equipment and equations used were extracted. The measurement techniques included in the study were: skinfold measurement; BIA; DXA; and ADP. Beside BF as the main variable of interest, lean compartment mass, including absolute (kg) or relative (%) muscle mass, fat free mass, or lean body mass were extracted and reported. For studies reporting multiple assessments (e.g. baseline, post-intervention, follow-up) of the same body composition indicator, the pre-intervention data or initial value were considered. Additional information regarding the ethical approval of studies, preparation for measurements (e.g. clothes, food intake, hydration) and reliability of results was also extracted. If pertinent data were absent, the authors were contacted, and the necessary information was requested via e-mail. In case of no response or unavailability of data, the article was excluded according to ineligibility criteria 5 (no data). Coding was cross-checked between authors and disagreements were settled by discussion until consensus was achieved.

### Data Synthesis and Presentation, Potential Effect Modifiers and Reasons for Heterogeneity

Statistical analysis was performed using R version 4.0.3, RStudio version 1.4.1103, and the package Metafor (version 3.0-2) [25]. The outcome variable was BF, and moderator variables were: sex (male, female); method of body composition assessment (ADP, BIA, DXA, and skinfold); and competitive level (international, national and regional) with random effect being the study itself. The pooled mean estimates, and their corresponding 95% confidence intervals (CI) were reported for each performed analysis. The variance of the sample mean BF for each study was calculated (SD^2^/sample size) and studies were weighted by the inverse of the variance in the meta-analysis models. The random-effects model takes into consideration the residual heterogeneity of studies and it is assessed through Cochran's test of heterogeneity (QE). In addition, *I*^2^ statistics were calculated to determine the degree of statistical heterogeneity, with > 75% considered as high statistical heterogeneity. Test statistics for residual heterogeneity by removing a single study were calculated to check for single study influence on residual heterogeneity. Sensitivity analysis was implemented to investigate the influence of the removal of a single study on the pooled estimate. Publication bias was visually inspected by examining the asymmetry of the funnel plots containing pseudo confidence interval regions (white (90%), light grey (95%) and dark grey (99%) areas). Forest plots were used to present pooled means with 95% CI of arbitrarily defined groups (e.g., male international players measured with DXA).

Each moderator variable was first considered independently (e.g. in a separate model including only one moderator). As the analysis demonstrated the statistically significant difference between groups in all single moderator variables (e.g., between females and males, between international and national/regional, and between measurement methods), we subsequently used the moderator sex in combination with another moderator (measurement method or competitive level). Finally, we combined all three moderators in one model. Hence, the model equation for the final model was

$$\hat{\theta }_{k} = \beta_{0} + {\text{female}}_{k} *\beta_{1} + {\text{BIA}}_{k} *\beta_{2} + {\text{Skinfold}}_{k} *\beta_{3} + {\text{ADP}}_{k} *\beta_{4} + {\text{national}}*\beta_{5} + {\text{regional}}_{k} *\beta_{6} + \zeta_{k} + \varepsilon_{k}$$

where $\hat{\theta }_{k}$ is the observed mean BF in study $k$, $\beta_{0}$ is the mean BF in the arbitrarily chosen reference group of male international players measured with DXA. Further regression coefficients $\beta_{1}$ to $\beta_{6}$ represent the change in mean BF due to female sex, measurement with BIA, skinfold or ADP, and national or regional competitive level. $\varepsilon_{k}$ is a residual term with mean 0 and variance corresponding to the sampling variance of $\hat{\theta }_{k}$ within the study-specific population of study $k$. $\zeta_{k}$ is an additional random effect with mean 0 and variance corresponding to the heterogeneity between studies.

Post-hoc Bonferroni correction was applied for *p*-values when performing all pairwise comparisons between the four methods of body composition assessment or the three competitive levels.

## Results

The search of the five databases resulted in a total of 2563 publications. After removal of duplicates, the titles and abstracts of 1305 studies were read. Following the application of the predetermined inclusion/exclusion criteria to both titles and abstracts, a total of 326 studies remained. Following further inspection of the full texts, 80 studies [2, 8, 9, 16, 18–20, 22, 26–97] were included into the meta-analysis (see Fig. 1).

A detailed summary of each of the included studies (authors and years of publication, populations, methods and outcomes) can be found in Tables 1, 2, 3 and 4. Across studies, 4335 basketball players were included (3467 male, 868 female) with a mean age ranging from 19.0 [22] to 28.9 [74] years. Mean body mass and body height ranged from 75.0 [28] kg to 105.6 [69] kg and 179.4 [48] cm to 203.0 [70] cm for males and 63.8 [67] kg to 81.1 [34] kg and 164.0 [67] cm to 185.8 [42] cm for females. Mean sample size was 55 players per study and ranged from 7 [74] to 1160 [16]. There were 652 players categorized as “regional level”, 2142 as “national level” and 1518 as “international”, with one study presenting a mixed sample of regional and national level players [91]. For the assessment of BF, 39 studies used skinfold measurements, 23 BIA, 15 DXA and 3 studies used ADP.

**Table 1:** Selected body composition parameters measured with dual-energy X-ray absorptiometry

Authors and year	Population						Methods		Outcomes	
	Sample size (*n*) and sex	Age (years) [mean ± SD or range]	Body mass (kg) [mean ± SD]	Body height (cm) [mean ± SD]	Level	Sample description	Equipment	Software	Body fat (%) [mean ± SD]	Lean compartment mass (kg) [mean ± SD]
Atalag et al. [26]^a^	36, M	21.3 ± 1.7	99.0 ± 16.2	191.4 ± 9.0	National	NCAA Division II	GE-Lunar Prodigy	enCORE v. 16.2	16.5 ± 4.4%	n.a
Dobrosielski et al. [27]	28, F	19.4 ± 1.3	73.1 ± 13.7	175.4 ± 9.3	National	NCAA Division I	GE-Lunar Prodigy	n.a	26.8 ± 4.5%	50.0 ± 6.9 kg [LBM]
Imeri & Dureha [28]	30, M	21.0 ± 1.7	75.0 ± 8.1	182.3 ± 9.2	National	Indian first league	n.a	n.a	15.3 ± 3.2%	63.4 ± 4.2 kg [LBM]
Nepocatych et al. [29]^a^	10, F	18—22	78.7 ± 16.8	n.a	National	NCAA Division I	GE-Lunar Prodigy	n.a	28.2 ± 7.6%	48.3 ± 5.1 kg [LBM]
Ploudre et al. [30]^a^	14, F	20.1 ± 1.2	72.1 ± 6.2	175.5 ± 5.7	National	NCAA Division II	GE-Lunar iDXA	n.a	28.5 ± 3.4%	49.1 ± 3.4 kg [LBM]
52.1 ± 3.8 kg [FFM]										
Raymond-Pope et al. [31]^b^	88, M	19.8 ± 1.3	95.2 ± 13.9	194.7 ± 9.1	National	NCAA Division I	GE-Lunar Prodigy	enCORE v. 16.2	13.8 ± 3.9%	76.5 ± 9.4 kg [LBM]
	122, F	19.9 ± 1.3	77.6 ± 13.3	177.9 ± 9.2					21.8 ± 9.7%	54.1 ± 6.7 kg [LBM]
Rockwell et al. [32]	14, M	19.9 ± 0.4	92.4 ± 4.1	191.8 ± 3.4	National	NCAA Division I	GE-Lunar Prodigy	Software version 8.10e	15.5 ± 0.8%	79.7 ± 2.8 kg [FFM]
	14, F	19.7 ± 0.3	69.7 ± 4.0	173.2 ± 3.4					23.7 ± 1.2%	49.3 ± 4.6 kg [FFM]
Sanfilippo et al. [33]	16, M	20.1 ± 1.5	92.1 ± 11.9	192.9 ± 8.9	National	NCAA Division I	GE-Lunar iDXA	enCORE v. 14.1	12.2 ± 2.0%	78.4 ± 8.3 kg [LBM]
	14, F	20.6 ± 1.6	76.3 ± 12.6	180.5 ± 7.0					23.6 ± 7.5%	54.6 ± 4.4 kg [LBM]
Sekel et al. [34]	11, M	20.4 ± 0.9	87.9 ± 12.5	188.6 ± 9.0	National	NCAA Division I	Hologic Horizon A	Hologic APEX Software v 5.5.3.1	15.0 ± 2.3%	n.a
	11, F	20.4 ± 1.0	81.1 ± 18.9	174.9 ± 9.0					25.5 ± 5.9%	n.a
Spiteri et al. [8]	12, F	24.3 ± 2.6	75.6 ± 14.6	177.7 ± 7.3	National	Australian WNBL	Hologic Discovery A	QDR for Windows, Hologic,		
v 12.4		28.1 ± 7.5%^c^	n.a							
Stanforth et al. [35]^a^	38, F	18–23	76.2 ± 0.8	178.6 ± 1.5	National	NCAA Division I	GE-Lunar Prodigy	enCORE v. 11.0	25.2 ± 0.5%	52.9 ± 0.3 kg [LBM]
Zanders et al. [36]	13, F	19.8 ± 1.3	74.6 ± 9.1	173.9 ± 13.6	National	NCAA Division II	Hologic QDR Discovery A	Hologic APEX Software		
v 4.5.3		27.1 ± 3.2%	52.8 ± 6.6 kg [FFM]							
Gantois et al. [37]	12, M	21.5 ± 2.7	80.6 ± 14.3	180.3 ± 1.6	Regional	Collegiate athletes	GE-Lunar Prodigy	n.a	23.0 ± 1.2%	60.5 ± 1.6 kg [FFM]
Taylor et al. [38]	14, F	20.4 ± 2.4^c^	78.7 ± 16.8^c^	169.6 ± 5.8^a^	Regional	NCAA Division III	Hologic Wi	n.a	25.3 ± 4.3%^a^	n.a
Sousa et al. [39]	10, M	23.3 ± 3.6	88.9 ± 15.3	192.9 ± 8.2	Regional	Brazilian state-level university league	GE-Lunar-DPX-NT	n.a	16.3 ± 8.0%	69.4 ± 6.9 kg [LBM]

*F* female; *FFM* fat free mass; *LBM* lean body mass; *M* male; *NCAA* National Collegiate Athletic Association; *n.a* not available*; SD* Standard deviation; *WNBL* Women’s National Basketball League

^a^Pre-intervention data or initial value of follow-up study presented; more data points available

^b^Studies that reported the data for different player’s position

^c^For studies not reporting pooled estimates for the sample mean and sample standard deviation, the respective values were calculated using the sample sizes (*n*1, *n*2), means (*m*1, *m*2) and standard deviations (sd1, sd2) reported for the individual groups. The according equations are pooled mean = (*m*1*n1 + *m*2*n2)/(*n*1 + *n*2) and pooled sample standard deviation = sqrt [(*n*1-1)*sd1^2 + (*n*2-1)*sd2^2 + *n*1*(*m*1-*m*)^2 + *n*2*(*m*2-*m*)^2) / (*n*1 + *n*2-1)]

**Table 2:** Selected body composition parameters measured with bioelectrical impedance analysis

Authors and year	Population						Methods		Outcomes	
	Sample size (*n*) and sex	Age (years) [mean ± SD or range]	Body mass (kg) [mean ± SD]	Body height (cm) [mean ± SD]	Level	Sample description	Equipment	Software	Body fat (%) [mean ± SD]	Lean compartment mass (kg or %) [mean ± SD]
Ljubojevic et al. [40]	26, F	21.2 ± 2.9^a^	74.0 ± 9.6 ^a^	182.0 ± 8.2 ^a^	International	Slovenian and Montenegrin players competing in WABA	Tanita BC-418 MA	n.a	20.1 ± 4.2% ^a^	33.0 ± 3.9 kg [MM] ^a^
Ljubojevic et al. [41]	27, F	23.6 ± 4.2^a^	73.9 ± 9.8 ^a^	182.1 ± 8.5 ^a^	International	Ukrainian and Montenegrin national team players	Tanita BC-418 MA	n.a	19.8 ± 4.6% ^a^	33.4 ± 3.8 kg [MM] ^a^
Mala et al. [42]	14, F	25.9 ± 4.2	76.6 ± 7.8	185.8 ± 9.0	International	Olympic silver medalist team	BIA 2000 M	n.a	21.2 ± 1.7%	60.3 ± 5.4 kg [FFM]
Vukašinović-Vesić et al. [43]	96, M	19.0 ± 0.8	90.6 ± 12.4	196.3 ± 8.2	International	National teams at U20 European championship	Tanita BC-418 MA	n.a	9.4 ± 3.8%	81.8 ± 9.5 kg [MM]
Delextrat et al. [44]	9, F	24.3 ± 4.1	65.1 ± 10.9	173.0 ± 7.9	National	English first league	Tanita, model n.a	n.a	21.1 ± 3.8%	n.a
Delextrat et al. [45]	8, M	23.0 ± 3.0	90.3 ± 9.6	190.5 ± 8.9	National	English University Premier League	Tanita, model n.a	n.a	12.8 ± 4.8%	n.a
	8, F	22.0 ± 2.0	77.6 ± 9.2	179.0 ± 8.5					22.5 ± 6.6%	n.a
Delextrat et al. [46]	9, M	22.8 ± 9.2	88.0 ± 10.3	191.3 ± 5.8	National	English second league	Tanita, model n.a	n.a	12.3 ± 4.6%	n.a
Delextrat et al. [47]	9, M	22.0 ± 3.0	90.9 ± 10.1	191.2 ± 8.5	National	English University Premier League	Tanita, model n.a	n.a	12.4 ± 4.7%	n.a
	8, F	21.0 ± 3.0	73.9 ± 9.7	176.4 ± 8.1					21.9 ± 5.5%	n.a
Delgado-Floddy et al. [48]	8, M	24.9 ± 2.7	89.7 ± 25.5	181.5 ± 8.4	National	Chilean second national division	BioSpace Inbody 120	n.a	19.3 ± 8.2%	46.3 ± 4.6% [MM]
Dzedzej et al. [49]^b^	14, M	n.a	94.4 ± 8.0	196.7 ± 8.7	National	Professional players with 20 ± 5.0 y experience	InBody 720	n.a	9.8 ± 4.0%	84.7 ± 8.7 kg [FFM] 49.1 ± 5.2 kg [MM]
Gryko et al. [50]^c^	35, M	24.5 ± 5.4	90.2 ± 10.5	193.4 ± 8.1	National	Poland first national league	Tanita BC-418	n.a	14.0 ± 3.1%	n.a
Köklü et al. [51]^c^	45, M	23.3 ± 3.9 ^c^	96.5 ± 13.4 ^c^	196.8 ± 8.2 ^c^	National	Turkish first and second leagues	Tanita BC-418	n.a	11.5 ± 4.4% ^c^	n.a
Kukric et al. [52]	60, M	n.a	94.6 ± 10.6	197.8 ± 8.1	National	Bosnia Herzegovina first league, Srpska first league	Tanita BC-418	n.a	12.0 ± 3.3%	n.a
Michalczyk et al. [53]	11, M	24.3 ± 2.6	91.4 ± 5.2	192.8 ± 3.6	National	Poland first national league, > 5 y experience	InBody 720	n.a	12.3 ± 2.4%	n.a
Michalczyk et al. [54]	15, M	23.5 ± 2.2	92.2 ± 5.2	194.3 ± 6.4	National	Poland first national league, > 5 y experience	InBody 720	n.a	12.4 ± 2.3%	79.6 ± 4.9 kg [FFM]
Ramos-Campo et al. [55]^c^	25, M	27.3 ± 1.2	96.1 ± 3.1	196.6 ± 1.9	National	Spanish first national league	InBody 720	Lookin’Body 3.0	12.8 ± 4.2% ^c^	49.8 ± 8.6 kg [MM]
Ribeiro et al. [9]	11, M	27.6 ± 4.9	89.6 ± 13.8	190.2 ± 10.1	National	Brazil national league	450 Bioimpedance Analyzer	n.a	13.6 ± 5.6%	76.4 ± 9.0 kg [FFM]
Stauffer et al. [56]^c^	13, F	19.7 ± 1.1	79.3 ± 18.2	179.0 ± 8.0	National	NCAA Division I	Tanita (model n.a.)	n.a	21.9 ± 5.3%	61.4 ± 11.4 kg [FFM]
Czuba et al. [57]	12, M	22.0 ± 1.9 ^a^	91.9 ± 12.1 ^a^	191.6 ± 6.7 ^a^	Regional	Polish well-trained athletes with > 5 y experience	InBody 720	n.a	11.1 ± 2.8% ^a^	n.a
Delextrat et al. [58]	15, F	23.3 ± 3.4	65.8 ± 6.3	173.1 ± 5.8	Regional	United Kingdom second University league	Tanita BC-418 MA	n.a	23.6 ± 4.9%	n.a
Delgado-Floddy et al. [48]	9, M	22.5 ± 3.8	83.6 ± 14.6	179.4 ± 8.0	Regional	Chilean collegiate league	BioSpace Inbody 120	n.a	16.6 ± 7.7%	47.6 ± 4.1% [MM]
Kutserib et al. [59]^b^	12, F	19.9 ± 1.6	64.0 ± 6.5	172.2 ± 8.1	Regional	Athletes with > 5 y experience	Tanita 400, Tanita BC-601	n.a	18.5 ± 3.7%	51.8 ± 6.5 kg [MM]
Omorczyk et al. [60]^b^	30, M	23.4 ± 1.8	84.4 ± 7.7	188.8 ± 5.8	Regional	Polish non-professional team	Tanita BC-582	n.a	12.5 ± 1.7%	71.7 ± 6.8 kg [LBM]
Salgueiro et al. [61]	11, M	19.9 ± 1.8	80.5 ± 10.1	180.0 ± 0.5	Regional	Brazilian Air Force Academy cadets	Tanita, BF-558	n.a	11.1 ± 3.0%	71.4 ± 8.6 kg [LBM]

*BIA; F* female; *FFM* fat free mass; *LBM* lean body mass; NCAA; *M* male; *MM* muscle mass; *n.a* not available; *SD* Standard deviation; *WABA* Women Adriatic Basketball Associaton; *y* years

^a^For studies not reporting pooled estimates for the sample mean and sample standard deviation, the respective values were calculated using the sample sizes (*n*1, *n*2), means (*m*1, *m*2) and standard deviations (sd1, sd2) reported for the individual groups. The according equations are pooled mean = (*m*1*n1 + *m*2*n2)/(*n*1 + *n*2) and pooled sample standard deviation = sqrt [(*n*1-1)*sd1^2 + (*n*2-1)*sd2^2 + *n*1*(*m*1-*m*)^2 + *n*2*(*m*2-*m*)^2) / (*n*1 + *n*2-1)]

^b^Pre-intervention data or initial value of follow-up study presented; more data points available

^c^Studies that reported the data for different player’s position

**Table 3:** Selected body composition parameters measured with skinfolds

Authors and year	Population						Methods		Outcomes	
	Sample size (*n*) and sex	Age (years) [mean ± SD or range]	Body mass (kg) [mean ± SD]	Body height (cm) [mean ± SD]	Level	Sample description	Equipment	Formula	Body fat % [mean ± SD]	Lean compartment mass (% or kg) [mean ± SD]
Ben Abdelkrim et al. [20]^a^	30, M	22.5 ± 4.6 ^b^	91.5 ± 7.6 ^b^	198.8 ± 6.7 ^b^	International	Tunisian senior and under-20 national teams	Lange	Durnin & Womersley (1974)	10.0 ± 2.4% ^b^	n.a
Albaladejo et al. [62]^ac^	8, M	26.8 ± 5.4	98.6 ± 15.4	198.4 ± 12.0	International	Spanish first and European leagues	Holtain	Rose & Guimaraes (1980)	11.2 ± 2.1%	48.0 ± 7.5 kg [MM]
Calleja-Gonzalez et al. [63]	27, M	24.9 ± 2.2 ^b^	95.8 ± 26.3 ^b^	196.2 ± 5.1 ^b^	International	Croatian and Japanese national teams	Harpenden	Jackson & Pollock (1980)	10.3 ± 1.8% ^b^	n.a
Cui et al. [16]^a^	1160, M	20.7 ± 1.4 ^b^	98.2 ± 11.6 ^b^	197.7 ± 8.5 ^b^	International	NBA drafted players	n.a	n.a	7.2 ± 2.7% ^b^	n.a
Doeven et al. [64]	14, M	26.7 ± 3.8	100.3 ± 15.2	197.2 ± 9.1	International	Dutch first and European leagues	n.a	n.a	10.3 ± 3.6%	n.a
Gonzalez et al. [65]	7, M	28.2 ± 3.4	104.7 ± 13.9	200.9 ± 9.4	International	NBA	n.a	Jackson & Pollock (1978)	7.2 ± 1.9%	n.a
Juric et al. [66]	28, M	24.0 ± 4.5	95.5 ± 12.3	194.5 ± 9.5	International	Croatian first and ABA leagues	Harpenden	Jackson & Pollock (1978)	12.7 ± 3.9%	n.a
Mtsweni et al. [67]	14, F	24.0 ± 3.5	71.1 ± 13.3	174.2 ± 1.0	International	South African national team	n.a	Durnin & Womersley (1974)	22.3 ± 5.5%	n.a
Ponce-González et al. [68]^a^	12, M	24.1 ± 4.9	91.8 ± 10.6	196.4 ± 10.1	International	Spanish first and Eurocup leagues	Holtain	Jackson & Pollock (1978)	10.3 ± 1.0%	n.a
Tsoufi et al. [69]^a^	15, M	24.0 ± 4.0	105.6 ± 18.2	203.0 ± 7.0	International	Greek first and European leagues	Harpenden	Jackson & Pollock (1978)	8.0 ± 2.7%	96.9 ± 14.6 kg [FFM]
Vaquera et al. [22]^a^	24, M	19.0 ± 0.1	93.4 ± 0.2	196.8 ± 1.9	International	Under-20 European championship	Harpenden	Juhasz (1974)	9.6 ± 2.4%	n.a
Zhao et al. [70]	16, M	21.2 ± 2.2	98.3 ± 9.9	203.0 ± 8.6	International	National and international competitions	Lange	Jackson & Pollock (1978)	14.4 ± 1.5%	n.a
Boone and Borgois [71]^a^	144, M	26.4 ± 5.3	95.9 ± 11.8	196.3 ± 7.2	National	Belgian first division	Harpenden	Parkizkova (1977)	12.9 ± 3.9%	n.a
Brini et al. [72]^c^	16, M	23.4 ± 2.3	78.3 ± 11.0	186.0 ± 9.0	National	Tunisian second division players	Harpenden	Durnin & Womersley (1974)	13.0 ± 1.7% ^b^	n.a
Chatzinikolaou et al. [73]	20, M	23.2 ± 2.5	92.9 ± 7.8	196.0 ± 5.1	National	Greek first league	n.a	n.a	9.8 ± 2.9%	n.a
Daniel et al. [74]	7, M	28.9 ± 6.0	94.8 ± 12.4	195.7 ± 6.7	National	Brazilian first league	n.a	n.a	11.1 ± 4.9%	n.a
Daniel et al. [75]	10, M	27.6 ± 5.5	91.6 ± 11.5	192.6 ± 7.6	National	Brazilian first league	Lange	Jackson & Pollock (1978)	10.7 ± 4.1%	n.a
Dragonea et al. [76]	12, M	26.0 ± 2.0	92.9 ± 2.5	191.0 ± 2.0	National	Greek first and second leagues	n.a	n.a	12.5 ± 1.1%	n.a
Ferioli et al. [19]^a^	67, M	25.5 ± 4.8 ^b^	94.6 ± 11.3 ^b^	197.6 ± 8.6 ^b^	National	Italian first and second league	Harpenden	Jackson & Pollock (1978)	11.3 ± 3.3% ^b^	n.a
Gomes et al. [77]^c^	11, M	25.1 ± 4.3 ^c^	101.5 ± 22.0 ^c^	195.4 ± 11.3 ^c^	National	Brazil national league	Lange	Jackson & Pollock (1978)	13.8 ± 3.6% ^b^	86.9 ± 16.4 kg [LBM]
Kariyawasam et al. [78]	30, M	24.0 ± 4.5	79.3 ± 12.9	183.3 ± 8.4	National	Sri Lankan national level players	Harpenden	Pollock & Jackson (1980)	11.6 ± 5.5%	n.a
Korkmaz et al. [79]	60, M	22.8 ± 4.0 ^b^	91.5 ± 10.9 ^b^	195.5 ± 5.2 ^b^	National	Turkish first and second division	Harpenden	Durnin & Womersley (1974)	13.6 ± 3.5% ^b^	78.8 ± 4.6 kg ^b^ [LBM]
asanovic et al. [80]	14, M	23.5 ± 2.8	99.6 ± 11.6	199.5 ± 7.4	National	Serbian first league	n.a	Matiegka (1921)	11.5 ± 2.0%	51.3 ± 2.0% [MM]
Pehar et al. [18]^a^	110, M	21.6 ± 3.9	89.3 ± 10.9	194.9 ± 8.1	National	Bosnian first and second league	Holtain	Durnin & Womersley (1974)	9.1 ± 3.4%	n.a
Peña et al. [81]	18, M	25.4 ± 5.2	92.6 ± 9.8	197.1 ± 8.8	National	Spanish first division	Holtain	Jackson & Pollock (1978)	7.4 ± 1.0%	n.a
Pireva [82]	130, M	19–35	91.5 ± 11.6	193.6 ± 7.8	National	Kosovan first league	Harpenden	Jackson & Pollock (1978)	15.7 ± 2.0%	76.9 ± 9.0 kg [LBM]
Pluncevic et al. [83]	110, M	20.9 ± 3.3	86.1 ± 12.3	191.2 ± 7.5	National	Macedonian premier league players	n.a	Matiegka (1921)	13.9 ± 4.0%	54.3 ± 3.7% [MM]
Puente et al. [84]	10, F	27.9 ± 6.1	70.9 ± 13.0	175.2 ± 0.1	National	Spanish second league	n.a	Carter (1982)	16.8 ± 5.4%	47.1 ± 4.3% [MM]
Sekulic et al. [85]^a^	110, M	21.6 ± 3.9	89.3 ± 10.9	194.9 ± 8.1	National	Bosnia and Herzegovina’s first and second divisions	Holtain	n.a	9.0 ± 3.4%	n.a
Thigpen et al. [86]	11, M	21.0 ± 1.0	85.4 ± 7.6	188.0 ± 9.0	National	NCAA Division II	Lange	Jackson & Pollock (1978)	8.7 ± 2.4%	n.a
	11, F	19.0 ± 1.0	75.3 ± 10.1	175.0 ± 8.0					14.2 ± 6.4%	n.a
Vaquera et al. [22]^b^	44, M	28.5 ± 1.2 ^b^	97.3 ± 3.1 ^b^	196.7 ± 2.9 ^b^	National	Spanish first and second leagues	Harpenden	Juhasz (1974)	11.9 ± 3.6% ^b^	n.a
Watson et al. [87]	9, F	19.9 ± 1.1	76.0 ± 10.3	176.0 ± 9.0	National	NCAA Division II	Lange	Jackson & Pollock (1985)	25.0 ± 2.7%	57.0 ± 7.6 kg [FFM]
De Oliveira et al. [88]^a^	183, M	26.4 ± 9.3	86.2 ± 21.6 ^b^	184.0 ± 11.1 ^b^	Regional	Brazilian state championship teams	Cescorf	Durnin & Womersley (1974)	17.9 ± 7.6% ^b^	n.a
Ferioli et al. [19]^a^	62, M	23.2 ± 5.7 ^a^	85.8 ± 12.8 ^a^	190.3 ± 8.5 ^a^	Regional	Italian third and fourth leagues	Harpenden	Jackson & Pollock (1978)	11.5 ± 4.2% ^a^	n.a
Freitas et al. [89]^c^	18, M	21.3 ± 4.3 ^b^	90.9 ± 14.8 ^b^	194.5 ± 11.4 ^b^	Regional	Spanish fourth league	Harpenden	Faulkner (1968)	12.3 ± 1.9% ^b^	44.4 ± 5.8 kg ^a^ [MM]
Gomez et al. [90]	44, M	18–30	89.1 ± 10.9	189.6 ± 8.1	Regional	Spanish university league	Holtain	Faulkner (1968)	14.9 ± 3.2%	47.7 ± 2.6% [MM]
	24, F	18–30	67.8 ± 8.3	173.0 ± 8.6					15.6 ± 2.3%	44.5 ± 2.9% [MM]
Korkmaz et al. [79]	30, M	22.1 ± 2.4	91.0 ± 8.6	195.0 ± 5.0	Regional	Turkish third division	Harpenden	Durnin & Womersley (1974)	12.1 ± 3.5%	78.1 ± 6.2 [LBM]
Marca [91]	18, M	25.0 ± 6.0	82.3 ± 8.8	183.1 ± 6.5	Regional	Spanish fourth and fifth league	n.a	n.a	15.9 ± 3.5%	43.8 ± 2.8% [MM]
	23, F	22.0 ± 3.0	67.4 ± 12.6	169.9 ± 9.5	Regional and National	Spanish second and third league			24.6 ± 5.9%	39.1 ± 5.5% [MM]
Masanovic et al. [76]	12, M	25.1 ± 3.2	90.6 ± 14.5	192.5 ± 4.6	Regional	Serbian fifth league	n.a	Matiegka (1921)	16.4 ± 6.8%	48.4 ± 3.6% [MM]
Mtsweni et al. [67]	41, F	21.7 ± 3.2 ^b^	63.8 ± 10.3 ^b^	164.0 ± 1.2 ^b^	Regional	South African university and provincial leagues	n.a	Durnin & Womersley (1974)	22.1 ± 5.8% ^b^	n.a
Pacheco Gabaldon et al. [92]	17, M	23.8 ± 5.6	87.3 ± 13.1	189.5 ± 9.1	Regional	Spanish federal league	Holtain	Carter (1982)	9.2 ± 1.9%	46.9 ± 1.6% [MM]
Puente et al. [84]	10, M	27.1 ± 4.0	89.5 ± 13.5	193.1 ± 8.8	Regional	Spanish fifth league	n.a	Carter (1982)	11.8 ± 3.1%	48.3 ± 2.4% [MM]
Scanlan et al. [93]	10, M	22.7 ± 6.1	86.5 ± 18.7	189.6 ± 9.5	Regional	Queensland Basketball League players	Harpenden	n.a	14.7 ± 3.5%	n.a
Scanlan et al. [94]	12, M	25.9 ± 6.7	97.4 ± 16.1	188.9 ± 7.9	Regional	Australian state-level basketball players	Harpenden	Evans et al. (2005)	15.5 ± 5.0%	n.a
Scanlan et al. [2]^a^	12, M	26.1 ± 5.3	85.9 ± 13.2	191.4 ± 7.6	Regional	Queensland Basketball League players	Harpenden	ACSM (2013)	11.5 ± 4.1%	n.a
	12, F	22.0 ± 3.7	72.9 ± 14.2	174.2 ± 7.0					17.2 ± 5.6%	n.a
Vaquera et al. [22]^a^	22, M	20.0 ± 0.8	89.7 ± 2.9	193.5 ± 2.0	Regional	Spanish fourth league	Harpenden	Juhasz (1974)	11.0 ± 4.7%	n.a

*ABA* Adriatic Basketball Association*; F* female; *FFM* fat free mass; *LBM* lean body mass; *NBA* National Basketball Association; *NCAA* National Collegiate Athletic Association; *M* male; *MM* muscle mass; *n.a* not available; *SD* standard deviation; *y* years

^a^Studies that reported the data for different player’s position

^b^For studies not reporting pooled estimates for the sample mean and sample standard deviation, the respective values were calculated using the sample sizes (*n*1, *n*2), means (*m*1, *m*2) and standard deviations (sd1, sd2) reported for the individual groups. The according equations are pooled mean = (*m*1*n1 + *m*2*n2)/(*n*1 + *n*2) and pooled sample standard deviation = sqrt [(*n*1 − 1)*sd1^2 + (*n*2-1)*sd2^2 + *n*1*(*m*1-*m*)^2 + *n*2*(*m*2-*m*)^2) / (*n*1 + *n*2-1)]

^c^Pre-intervention data or initial value of follow-up study presented; more data points available

**Table 4:** Selected body composition parameters measured with air displacement plethysmography

Authors and year	Population						Methods			Outcomes	
	Sample size (*n*) and sex	Age (years) [mean ± SD or range]	Body mass (kg) [mean ± SD]	Body height (cm) [mean ± SD]	Level	Sample description	Equipment	Software		Body fat (%) [mean ± SD]	Lean compartment mass (kg) [mean ± SD]
Fields et al. [95]^a^	127, M	18–24	94.5 ± 12.6^b^	194.0 ± 9.2^b^	National	NCAA Division I	Bod Pod 2000A, COSMED	n.a		11.5 ± 5.1^b^	83.2 ± 8.5 kg [FFM]
	196, F	18–24	74.6 ± 12.3^b^	177.1 ± 7.6^b^						21.5 ± 6.5^b^	57.9 ± 6.3 kg [FFM]
Fields et al. [96]	95, F	18–22	73.6 ± 11.9	177.9 ± 7.6	National	NCAA Division I	Bod Pod 2000A, COSMED	n.a		21.6 ± 6.7	57.2 ± 6.1 kg [FFM]
Ladwig et al. [97]^c^	11, F	20.1 ± 1.8	71.1 ± 10.9	176.5 ± 8.3	National	NCAA Division I	Bod Pod 2007A, COSMED	n.a		22.3 ± 5.5	75.5 ± 13.1 kg [FFM]

*F* female; *FFM* fat free mass; *NCAA* National Collegiate Athletic Association; *M* male; *n.a* not available; *SD* Standard deviation

^a^Studies that reported the data for different player’s position

^b^For studies not reporting pooled estimates for the sample mean and sample standard deviation, the respective values were calculated using the sample sizes (*n*1, *n*2), means (*m*1, *m*2) and standard deviations (sd1, sd2) reported for the individual groups. The according equations are pooled mean = (*m*1*n1 + *m*2*n2)/(*n*1 + *n*2) and pooled sample standard deviation = sqrt [(*n*1 − 1)*sd1^2 + (*n*2 − 1)*sd2^2 + *n*1*(*m*1-*m*)^2 + *n*2*(*m*2-*m*)^2) / (*n*1 + *n*2-1)]

^c^Pre-intervention data or initial value of follow-up study presented; more data points available

Our results revealed that male players had significantly lower BF values compared to their female counterparts (pooled mean for males = 13.2%; 95% CI 12.4–14.0% vs. pooled mean for females = 20.4%; 95% CI 19.4–21.3%; *p* < 0.001). BF measured by DXA (pooled mean = 21.6%; 95% CI 18.5–24.7%) was significantly higher than BF measured by BIA (pooled mean = 14.7%; 95% CI 12.2–17.3%; *p* < 0.001) and skinfolds (pooled mean = 12.3%; 95% CI 10.4–14.2%; *p* < 0.001). Furthermore, BF measured by skinfolds was significantly lower than BF measured by ADP (pooled mean = 20.0%; 95% CI 13.3–26.6%, *p* = 0.02). Pooled mean BF values across competitive levels were 13.2% for international level players (95% CI 11.3–15.1%), 15.6% for national level players (95% CI 14.0–17.1%) and 15.0% for regional level players (95% CI 13.3–16.6%), with a significant difference found between international and national level players (*p* < 0.001) as well as international and regional level players (*p* = 0.02).

**Fig. 2:**
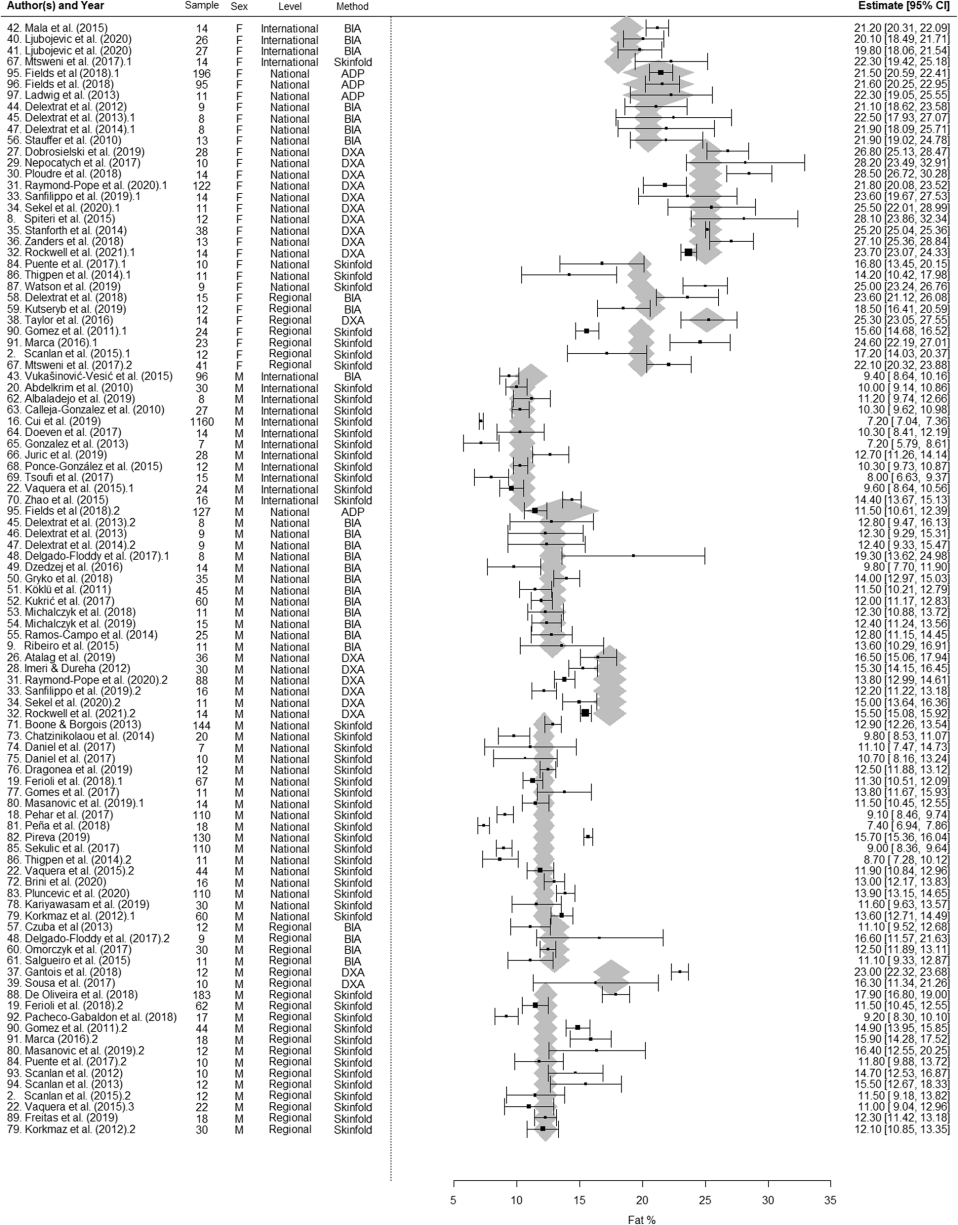
Relative body fat of basketball players: forest plot showing pooled mean estimates and 95% confidence intervals of included studies. *ADP* air displacement plethysmography; *BIA* bioelectrical impedance analysis; *CI* confidence interval; *DXA* dual-energy X-ray absorptiometry; *F* female; *M* male; 1, 2, 3 single study included multiple times in the forest plot as it included data from multiple samples (e.g. male and females; international and regional); * marking different studies from same authors and published in the same year

A random-effects meta-regression model was used to examine the effects of sex, measurement method and competitive level on BF. Our model combining all variables revealed that BF differences between male and female players stayed significant (*p* < 0.001) after correcting for competitive level and measurement method. Similarly, the differences between BF as measured by DXA and BIA as well as by DXA and skinfold remained significant (*p* < 0.001) after accounting for sex and competitive level. By contrast, the differences between BF measured by ADP and skinfolds were no longer significant after adjusting for sex and competitive level. Differences between international players and national players (*p* = 0.02) as well as differences between international and regional players (*p* = 0.02) remained significant after adjusting for sex and measurement method. However, sensitivity analysis suggested that the analysis of the influence of competitive level was not completely robust. Exclusion of one study [18] changed the statistical significance. By contrast, the stability of our findings on measurement method and sex were confirmed by the sensitivity and cumulative meta-analyses. The forest plot of the analysis is presented in Fig. 2. The results of meta-analysis according to subgroups adjusted for sex and measurement method are shown in Table 5.

**Table 5:** Results of meta-analysis according to sex and measurement method

Method	Sex	No of studies	No of subjects	Body fat (%)^a^	
				Mean (pooled)	95% CI
DXA	M	8	217	17.5	15.4–19.5
	F	11	290	25.0	23.0–27.1
BIA	M	16	408	12.4	10.6–14.2
	F	9	132	20.2	18.5–21.8
Skinfolds	M	37	2715	11.7	10.5–12.9
	F	6	144	19.3	17.9–20.7
ADP	M	1	127	13.7	9.3–18.1
	F	3	302	21.3	16.9–25.7

*ADP* air-displacement plethysmography; *BIA* bioelectrical impedance analysis, *CI* confidence interval; *DXA* dual-energy X-ray absorptiometry; *F* female, *M* male

^a^Body fat values presented are pooled estimates and 95% confidence intervals adjusted for sex and measurement methods

**Fig. 3:**
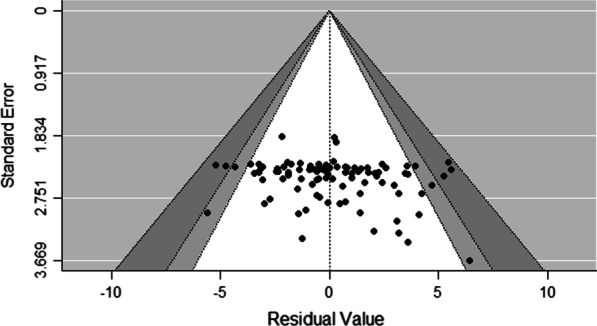
Funnel plot of the model including all moderator variables

We found no indication of a publication bias, with most points falling symmetrically within the funnel plot (see Fig. 3). Heterogeneity in our dataset was estimated by Cochran's test of heterogeneity (QE = 2621, *p* < 0.0001) and *I*^2^ statistics (*I*^2^ > 75%). The Cochran's test of heterogeneity revealed highly stable outcomes in our case when we ran a sensitivity analysis for *p*-values by removing single studies step-by-step (i.e., no changes in *p*-values).

## Discussion

This is the first systematic review and meta-analysis to examine body fat in basketball players as well as the respective influences of sex, measurement method and competitive level. The main findings of this meta-analysis were: (1) male basketball players have greater BF compared to their female counterparts; (2) considerable differences exist between BF as assessed with different methods, with greater BF values reported from DXA analysis compared to BIA and skinfold estimates; and (3) BF is lower in international level players compared to lower level (i.e. national and regional) players. In general, the BF data obtained by our meta-analysis (see Table 5) are in a healthy, athletic-level range. Aside from this general outcome, as all the factors investigated significantly influenced BF, it is essential to discuss and interpret results in consideration of the player’s sex, competitive level and the measurement method implemented.

Given the increasing popularity of women’s basketball and the general need for more high-quality sports science research focusing on female athletes [98], the present study made a particular effort to evaluate the effect of sex on BF of basketball players by including sex as a potential factor into the meta-regression. As initially expected, BF values were greater in female basketball players than in males. These results were confirmed even when considering the moderating effects of measurement method and competitive levels. While a previous direct comparison across male and female basketball players has shown similar results [2], our study compiled all previous relevant research on body composition of basketball players. Females carry greater BF than males due to biological differences [15] which have to be taken into account by practitioners working with female basketball players, from both performance (e.g. speed, power training) and health (e.g. manipulating training loads to reduce risk of injury) perspectives. Despite the increasing number of publications focusing on female basketball players in recent years, the body of evidence available on women is still much smaller than that available for men (3467 male players included versus 868 female players). Considering the already comparatively low number of female athletes included into this meta-analysis, it should be noted that only 8 of the 44 studies involving female athletes estimated BF content through measurements of skinfold thickness. Hence, the respective reference values reported here must be interpreted carefully. While skinfold assessment has some limitations [99], it is also the least expensive method and most frequently used by practitioners [99]. For these reasons, further research into the anthropometry of female basketball players is warranted to obtain more robust reference data.

Interestingly, considerable differences were found between BF values registered with different measurement methods. BF as measured by DXA was significantly higher compared to BF measured by BIA or skinfolds. Thus, our meta-analysis confirms the results of a single original study, in which BF values measured by different methods were compared in the same sample [30]. Furthermore, it has been observed by various studies that athletes` BF measured by skinfold or BIA is significantly underestimated when directly compared to BF measured by DXA [100, 101]. Given these differences, it is recommended to compare BF values only to reference values derived with the same measurement method (see Table 1, 2, 3, 4, 5). Additionally, results can also be affected by measurement preparation as well as the type of measurement equipment and the computational procedures used for the estimation of BF content [17, 102]. As an example, Golja et al. [102] observed that BF estimates of young, healthy subjects ranged from 6 to 29% across several skinfold regression equations. Similarly, large variability between measurement devices and equations have been found for BIA and DXA derived values of body composition [17, 103]. This carries important implications for practitioners assessing BF levels in athletic cohorts and comparing their results to data reported in the literature. If possible, data should be compared to values obtained with the same measurement equipment and computational procedure. Equally, it is imperative that future studies clearly state both measurement devices and computational procedures. Another important point to consider is measurement methodology standardization. Even though it is well known that factors such as hydration status, food intake, physical activity and temperature can influence all body composition measurement methods [17, 103, 104] about half of the studies included in this review did not provide adequate details regarding measurement methodology standardization. Another secondary finding that might help future research planning is that only about one third of the studies included in this review reported measures of reliability (e.g. coefficient of variation, intraclass correlation coefficient, etc.) for their body fat assessments. However, this is important to ensure that data are sound, and results are accurate.

Regarding competitive levels, we found BF levels to be significantly lower in international-level players compared to national or regional players. However, it should be noted that the sensitivity analysis of the data showed that findings were influenced by single studies, which means caution is needed in their interpretation. While we expected to find lower BF values in higher competitive levels, differences between groups were generally small and could be only observed when comparing the international to lower competitive levels. While lower BF is advantageous for neuromuscular actions such as jumps and changes of directions [8, 9], the game of basketball is also characterised by static efforts. These actions refer to all those situations in which players are stationary and fight to obtain and maintain advantageous position on the court (e.g.to rebound, in picking and low-post situations) [3, 105]. In these specific scenarios, a greater body mass might be advantageous for the player, making him/her less prone to be pushed away from his/her position by an opponent. Since previous studies have shown that higher level players have a greater body mass than lower-level players [19, 20, 22], it is possible that lean compartment mass, rather than BF, is more sensitive in discriminating between basketball players of different competitive levels. While we extracted lean compartment mass from all included studies (see Tables 1, 2, 3, 4), inconsistencies in terminologies and calculation methods used impeded their joint evaluation by meta-analysis. Future studies should address these inconsistencies and clearly state how lean compartment mass was calculated. Nevertheless, our results evidenced that BF content was lower in higher competitive levels in basketball, an expected finding which might be explained by several factors related to competing at higher levels, such as more rigorous anthropometric profiling and selection processes, controlled diet, as well as higher physical, physiological and energetic demands of training and competition.

This study had some limitations. Firstly, most studies did not report reliability measures of the body composition methods implemented, which casts doubt on the reproducibility of included data. Similarly, few studies reported essential information such as hydration and feeding status—factors known to influence body composition measurements [17, 104]. Another limitation regarded the categorisation of competitive level, which could also have influenced our results. We categorised players as international, national or regional, but this classification may improperly reflect the players’ actual competitive or skill level (e.g., the competitive level in a regional league in the U.S. might actually be higher than that in a national league of a country where basketball is less popular). Lastly, since only 19 out of 80 included studies reported BF values by playing position, it was not possible to account for playing position in the present meta-analysis. Players of different positions typically feature significantly different anthropometric characteristics and performance profiles [3, 20], so there is a clear need for future studies to report BF data by playing position.

This study also aimed at critically discussing the shortcomings of research published to date, and to identify promising future research directions. We recommend future studies assessing BF of basketball players to (1) clearly describe computational procedures and measurement devices used to estimate BF (2) specify the reliability of the measurement instruments, (3) clearly control and report the hydration and feeding status prior to measurement, (4) specify the competitive level of the sample by reporting the country and/or region and name of the league in which players competed at the time of the study, and (5) report BF of players in distinct categories (i.e. sex, competitive level, playing position) for better interpretation of data. Additionally, it would be interesting to review the influence of sex, measurement method and competitive level on lean compartment mass values, such as fat free mass, lean body mass and muscle mass. However, inconsistencies in terminology could be an important barrier to the successful (quantitative) comparison of studies investigating lean compartment mass of basketball players.

## Conclusion

This meta-analysis summarised and evaluated the available body of evidence on BF of basketball players. The results showed that female basketball players have greater BF than male counterparts. Results for the same basketball players varied depending on the measurement method used; therefore, it is imperative for practitioners assessing BF to compare their players’ BF only with the values obtained in this study for the same measurement method. International-level players appeared to have lower BF than national or regional level players, suggesting that body composition variables can discriminate competitive levels in basketball.

## Data Availability

Data will be made available upon reasonable request.

## Abbreviations

ADP: Air displacement plethysmography
BF: Body fat
BIA: Bioelectrical impedance analysis
95% CI: 95% Confidence interval
DXA: Dual-energy X-ray absorptiometry
NBA: National Basketball Association
NCAA: National Collegiate Athletic Association
QE: Cochran's test of heterogeneity
SD: Standard deviation
